# Disruption of *Drosophila* larval muscle structure and function by UNC45 knockdown

**DOI:** 10.1186/s12860-021-00373-7

**Published:** 2021-07-13

**Authors:** Abiramy Karunendiran, Christine T. Nguyen, Virginijus Barzda, Bryan A. Stewart

**Affiliations:** 1 Department of Cell and Systems Biology, University of Toronto, Toronto, ON Canada; 2 Department of Physics, University of Toronto, Toronto, ON Canada; 3 Department of Chemical and Physical Sciences, University of Toronto Mississauga, Mississauga, ON Canada; 4 Department of Biology, University of Toronto Mississauga, Mississauga, ON Canada

## Abstract

**Background:**

Proper muscle function is heavily dependent on highly ordered protein complexes. UNC45 is a USC (named since this region is shared by three proteins UNC45/CRO1/She4P) chaperone that is necessary for myosin incorporation into the thick filaments. UNC45 is expressed throughout the entire *Drosophila* life cycle and it has been shown to be important during late embryogenesis when initial muscle development occurs. However, the effects of UNC45 manipulation at later developmental times, after muscle development, have not yet been studied.

**Main results:**

UNC45 was knocked down with RNAi in a manner that permitted survival to the pupal stage, allowing for characterization of sarcomere organization in the well-studied third instar larvae. Second harmonic generation (SHG) microscopy revealed changes in the striated pattern of body wall muscles as well as a reduction of signal intensity. This observation was confirmed with immunofluorescence and electron microscopy imaging, showing diminished UNC45 signal and disorganization of myosin and z-disk proteins. Concomitant alterations in both synaptic physiology and locomotor function were also found. Both nerve-stimulated response and spontaneous vesicle release were negatively affected, while larval movement was impaired.

**Conclusions:**

This study highlights the dependency of normal sarcomere structure on UNC45 expression. We confirm the known role of UNC45 for myosin localization and further show the I-Z-I complex is also disrupted. This suggests a broad need for UNC45 to maintain sarcomere integrity. Newly discovered changes in synaptic physiology reveal the likely presence of a homeostatic response to partially maintain synaptic strength and muscle function.

## Background

Muscle function depends on the proper assembly of the sarcomere. Current models show that sarcomere assembly begins with the development of the Z-discs [1], recruiting proteins such as titin and α-actinin to serve as a scaffold for the integration of thin filaments to form the I-Z-I complex [2–4]. The thin filament develops through actin polymerization and troponin integrates with the developing filaments in order to recruit tropomyosin [5]. Myosin filaments are later integrated into this scaffold [2, 6]. For each step of sarcomere assembly, molecular chaperones are responsible for incorporating its targeted protein with proper folding [7]. In addition, after initial development, myosin and actin filaments are constantly synthesized and integrated into the muscle fiber. This demanding process requires a variety of molecular chaperones that both aid in the integration of actin and myosin molecules and prevent aggregation of misfolded proteins [8]. With myosin filaments, chaperone activity is especially important during the late embryo stages of *Drosophila*, as well as late pupal stages since the majority of the muscle development occurs in these stages.

One chaperone that is specifically vital for myosin folding, transport and integration is UNC45 [9, 10]. UNC45 is a chaperone that interacts with myosin II and myosin V [11]. In both vertebrates and invertebrates UNC45 is composed of three domains [7, 12]: an N-terminal tetratricopeptide repeat (TPR) domain that interacts with Heat Shock Protein 90 [12], a central domain with an unknown function and a UCS domain (aptly named since this region is shared by three proteins (UNC45, CRO1 and She4P)) which interact with myosin and facilitate myosin function [13]. Changes in UNC45 levels or expression pattern have been shown to negatively affect myosin function in muscle [9, 14, 15]. Thus, UNC45 has been of keen interest in studying muscle development.

Somatic muscles in the *Drosophila* in the larval body wall are a well-studied muscular system, but the degree that UNC45 contributes to muscle maintenance, stability or function has not been completely elucidated. The relatively few studies to date have focused primarily on early development of embryonic muscles or on targeted experiments on cardiac muscle. Investigations using UNC45 mutations have shown severe disturbance in sarcomere structure during muscle development, leading to embryonic lethality [10]. Knockdown experiments specifically targeting heart muscles showed severe reduction in muscle contractility and disassembled cardiac myofibrils in both larval and pupal stages [9]. In our previous work, we have focused on the subcellular structure and function of muscle [16–18], with particular focus on harmonic imaging of healthy larval muscle. In the present study we begin examination of muscle carrying mutations with the potential to disrupt normal structure and function. Given its role in myofibril assembly we chose to study UNC45 knockdown targeted to larval body wall muscle. Using harmonic and immunofluorescence imaging we demonstrate severe disruption of muscle structure in third instar larvae, alongside impaired synaptic transmission and locomotory behaviour. These findings will allow for more detailed analysis of muscle structure when it is in a dysfunctional state using harmonic microscopy.

## Results

*Drosophila* larvae have eight abdominal segments, with striated body wall muscles attached to the inner cuticular surface. Each hemi-segment contains 30 unique, multinucleated, single-cell muscles. The genetic tractably of this organism along with muscle identifiability, relative ease of imaging, and conservation of molecular components make *Drosophila* an ideal system for the study of development and physiology of muscle. In order to assess the contribution of UNC45 to muscle development and function, we first tested knockdown of UNC45 with three widely used muscle driver lines in the UAS-Gal4 expression system. Knockdown of UNC45 via UAS-RNAi constructs crossed to mef2-Gal4 and 24B-Gal4 were both lethal at early developmental stages, whereas viable larvae were obtained with c57-Gal4. The progeny from this cross were however slower to develop on standard *Drosophila* media, taking 7 days to develop to third instar larvae, instead of 5 days like the wildtype. In addition, we noted late pupal lethality as no adults emerged from the pupal cases. The UAS-UNC45-RNAi line was previously validated by Western blot to reduce UNC45 expression [9].

### Second harmonic imaging following UNC45 knockdown

**Fig. 1:**
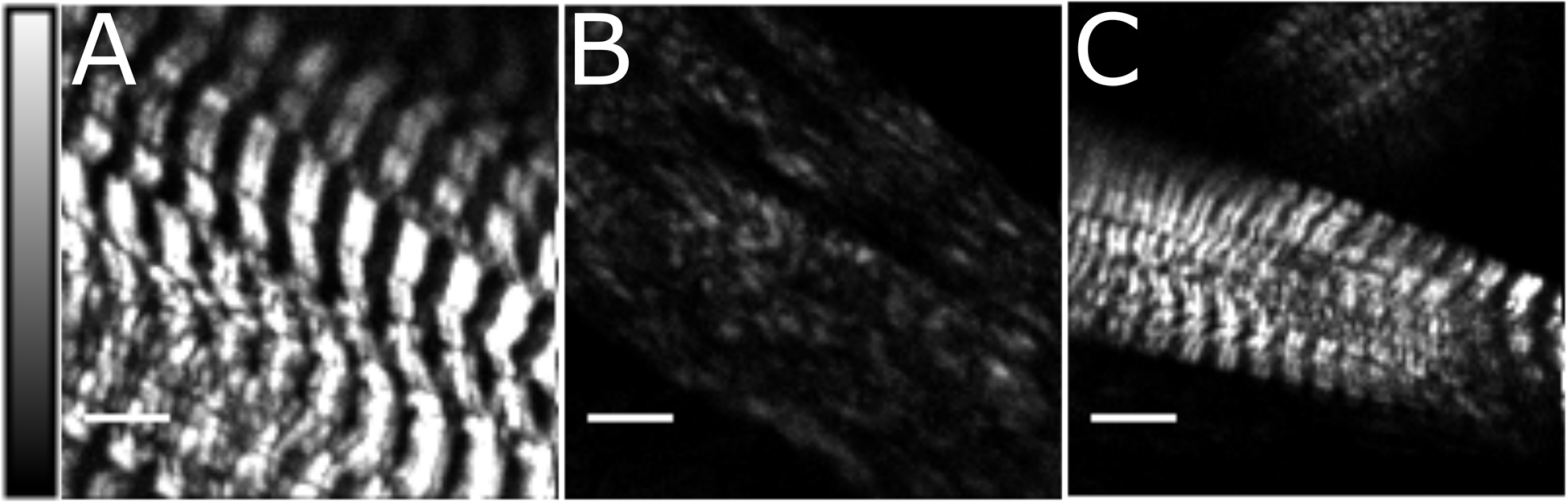
SHG images of UNC45 knockdown muscles in 3rd instar larva. These are representative images of **A**) wildtype, **B**) UNC45 knockdown with severe disruption in the striated pattern and **C**) UNC45 knockdown muscles with striations. SHG intensity can be compared since samples are imaged under identical imaging parameters. Scale bars represent 10 μm. Intensity scale bar indicates SHG signal in each pixel

In order to obtain a general understanding of muscle structure in UNC45 knockdown larvae, tissues were examined using Second Harmonic Generation (SHG) microscopy (Fig. 1). SHG microscopy is a laser scanning imaging technique that has been used to image a variety of biological samples without the need for staining protocols. The SHG process requires ordering of non-centrosymmetric molecules for the signal to be generated; myosin filaments in striated muscle have been extensively studied using this method [16, 19–21]. SHG microscopy provides a good representation of the changes in muscle structure shown previously with the use of myosin mutants [18, 22] and it can suggest defects that can be further analyzed using antibodies. Wild-type larvae show a very typical banding pattern with SHG imaging, characteristic of these striated muscles (Fig. 1A). In UNC45 knockdown larvae we observed a severe disruption in the banding pattern and reduced SHG signal intensity in many muscles (Fig. 1B). However, there was incomplete penetrance of this phenotype: some muscles where severely affected with almost no signal evident, other muscle had weak or partial banding and few individual muscles showed near normal banding (Fig. 1C). The degree of phenotypic penetrance is documented further below.

In order to perform accurate SHG intensity comparisons, UNC45 knockdown and wildtype control samples were imaged on the same day, under identical laser power, frame size and acquisition time. Compared to SHG intensity of the wildtype (178 ± 172 A.U.: Fig. 1A), UNC45 muscles with alteration in the sarcomere structure have a significantly lower second harmonic response (33 ± 29 A.U.: Fig. 1B). UNC45 muscles that exhibited some striated patterns were found to be more similar in intensity compared to the control (173 ± 100 A.U.: Fig. 1C). Similar trends were seen across multiple animals (*n* = 10). These intensity differences in UNC45 knockdown muscle suggest a major reorganization of the crystalline structure required to observe SHG signal.

### Changes in muscle structure

**Fig. 2:**
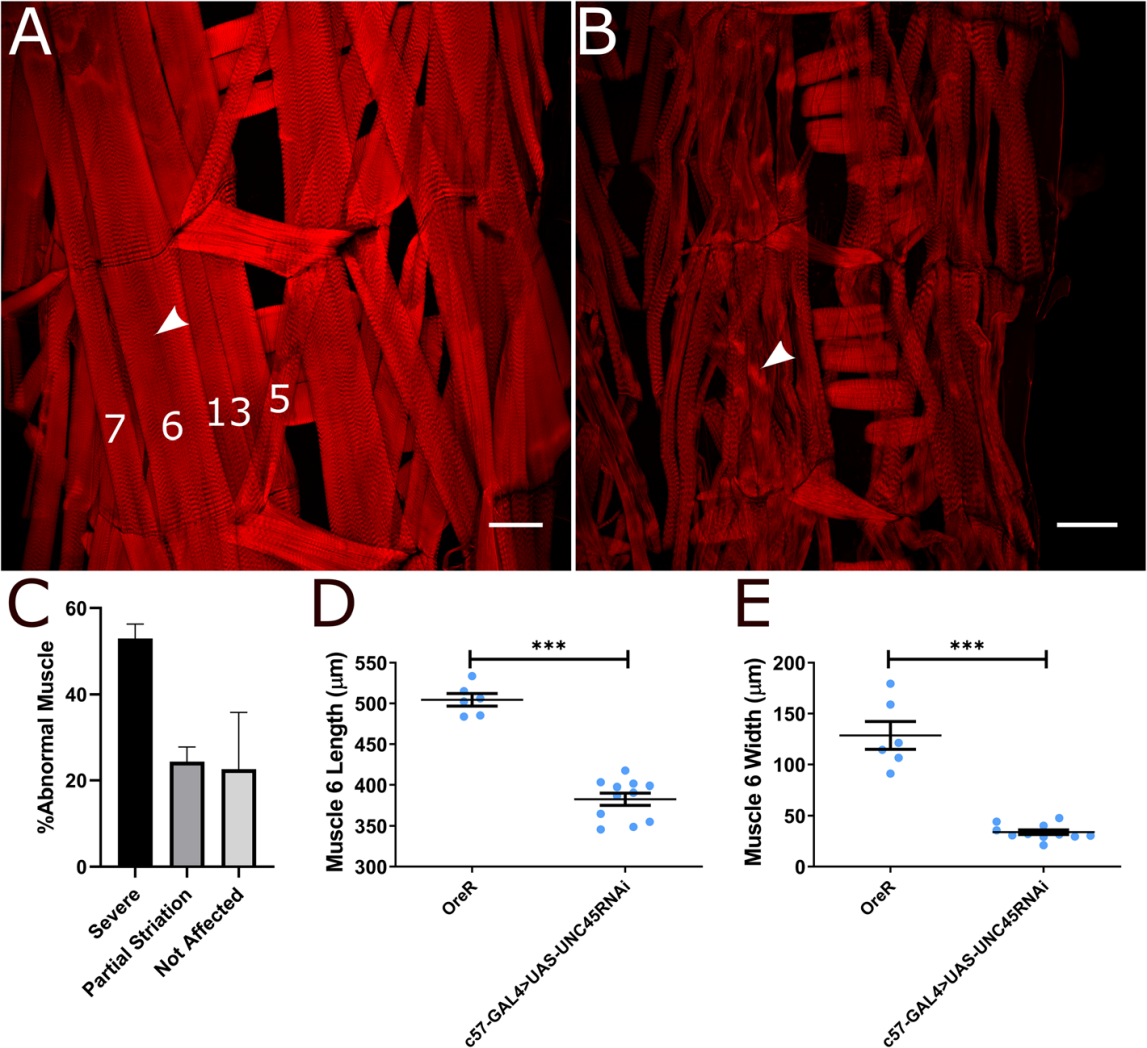
Initial fluorescence images of UNC45 knockdown muscles in 3rd instar larvae. These are representative hemi-segment images of **A**) wildtype and **B**) c57 > UNC45RNAi body wall muscles. Arrowhead indicates muscle 6. **C**) Graph depicting the frequency of abnormal muscle patterning in c57 > UNC45RNAi muscles (*n* = 8). **D**) Average length of muscle 6. **E**) Average width of muscle 6 indicated by the arrow in **A**) (*n* = 10). Muscles 5, 6, 7 and 13 are label in **A**). Plots show mean ± SEM. Scale bar in **A** and **B** represents 100 μm

We next took a more traditional approach to examine expression and localization of molecular components of the sarcomere. Actin was imaged by way of phalloidin staining and we observed that compared to the wildtype (Fig. 2A) there was a major disruption in the striated pattern in UNC45 knockdown muscle, corroborating our SHG imaging (Fig. 2B). Though actin filaments were observed to be aligned along the long axis of the muscle fiber, most muscles in UNC45 knockdown larvae did not show the normal phalloidin banding pattern. Furthermore, as documented with SHG imaging, we observed variability in the penetrance of the phenotype. Many individual muscles were severely affected where there was no banding pattern observed, while some remained partially striated where the banding pattern was either partially disrupted or discontinuous towards the center of the muscle fiber, and a few muscles appearing normal.

From the phalloidin stained samples, we quantified the degree of phenotypic penetrance by categorizing and counting the number of muscles affected within a hemi-segment. (Fig. 2C). We observed that on average 50% of the muscles in a hemi-segment were severely affected and did not show any striations, 25% of the muscles were observed to have partial banding pattern at the ends of the fiber that was weaker or absent towards the middle muscle or have a highly irregular striations along the muscle, while 25% showed a near normal pattern.

Wildtype larvae always showed 100% strong banding. Although certain muscles were not always affected in each animal by the knockdown, we observed that muscles 5, 6, 7 and 13 were affected in every sample analyzed. The knockdown larvae were found to be smaller and thinner than the wild-type control. The most severely affected muscles, that exhibited little to no striations, were also observed to be shorter and thinner than the wild type control. Measurements of muscle 6 (indicated by the arrow in Fig. 2A) length (Fig. 2D) and width (Fig. 2E) revealed that UNC45 knockdown muscle is significantly shorter (382 ± 24 μm) and thinner (34 ± 7 μm) compared to muscle 6 in wildtype larvae (504 ± 18 and 128 ± 33 μm respectively).

### Changes in sarcomere structure

**Fig. 3:**
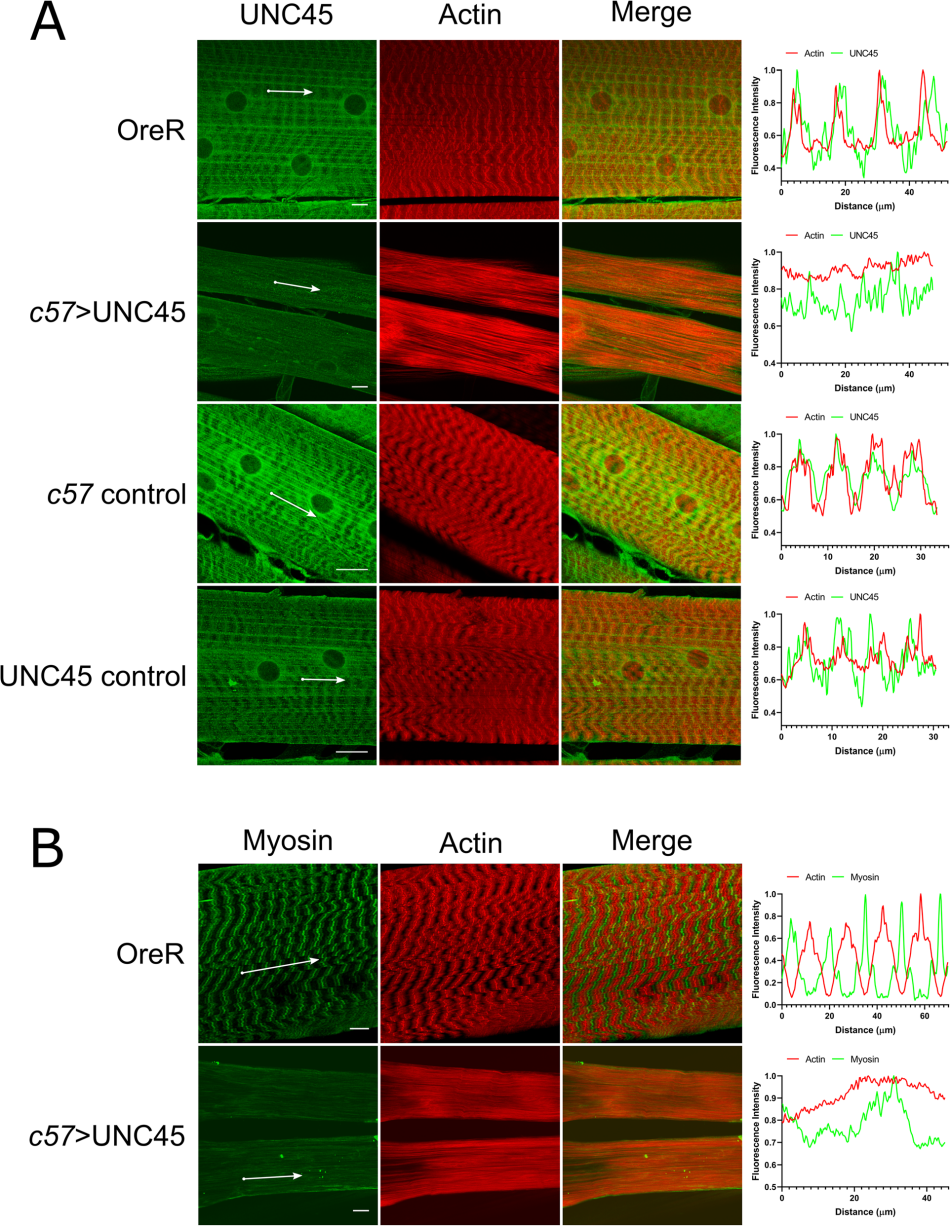
Changes in UNC45 and myosin localization in UNC45 knockdown larvae. Body wall muscles were labelled for **A**) actin (phalloidin) and UNC45 or **B**) actin and myosin. For each, the top panel shows the wildtype control animal and bottom panel show the c57 > UNC45 animals. Images show muscle 6. Fluorescence intensity profiles on the right were taken along muscle fibrils indicated by the white arrows in the images. Scale bar represents 20 μm

UNC45 knockdown experiments done previously for larval heart development have shown that there is a decrease in both UNC45 and myosin expression levels through western blot analysis [9]. In order to visualize the RNAi-induced decrease in UNC45 and to examine myosin distribution in third instar larval muscles, immunofluorescence was performed (Fig. 3). First, UNC45 and actin localization was tested with double immunofluorescence imaging (Fig. 3A). In wildtype muscles UNC45 localized in the same spatial regions as phalloidin-labelled actin, identifying the I-bands of the muscle, consistent with previous reports. Fluorescence intensity profile analysis shows the peaks and troughs of fluorescence marking muscle sarcomeres and the in-phase signals of actin and UNC45 labels. Genetic controls for *c57-*Gal4 and UAS-UNC45 RNAi were also observed to have similar fluorescence profiles to the wild-type.

We next analyzed double immunofluorescence analysis of actin and myosin (Fig. 3B) which showed the expected out-of-phase banding pattern of fluorescence intensity. In c57-Gal4 > UAS-RNAi tissues we observed severe disruption of UNC45, actin and myosin signals. In a qualitative confirmation that the RNAi was effective, we observed much reduced UNC45 antibody labelling; RNAi expressing samples were processed and imaged under identical conditions as control tissue. Additionally, in the UNC45 knockdown muscles, the myosin fluorescence signal was likewise reduced. In the severely affected muscles, there was a loss of the normal banding pattern and none of the labels for UNC45, actin, or myosin were observed to localize in their respective spatial regions as seen in wildtype muscles. We did however observe a significant actin signal, appearing to represent actin filaments in parallel to the long axis of the muscle.

**Fig. 4:**
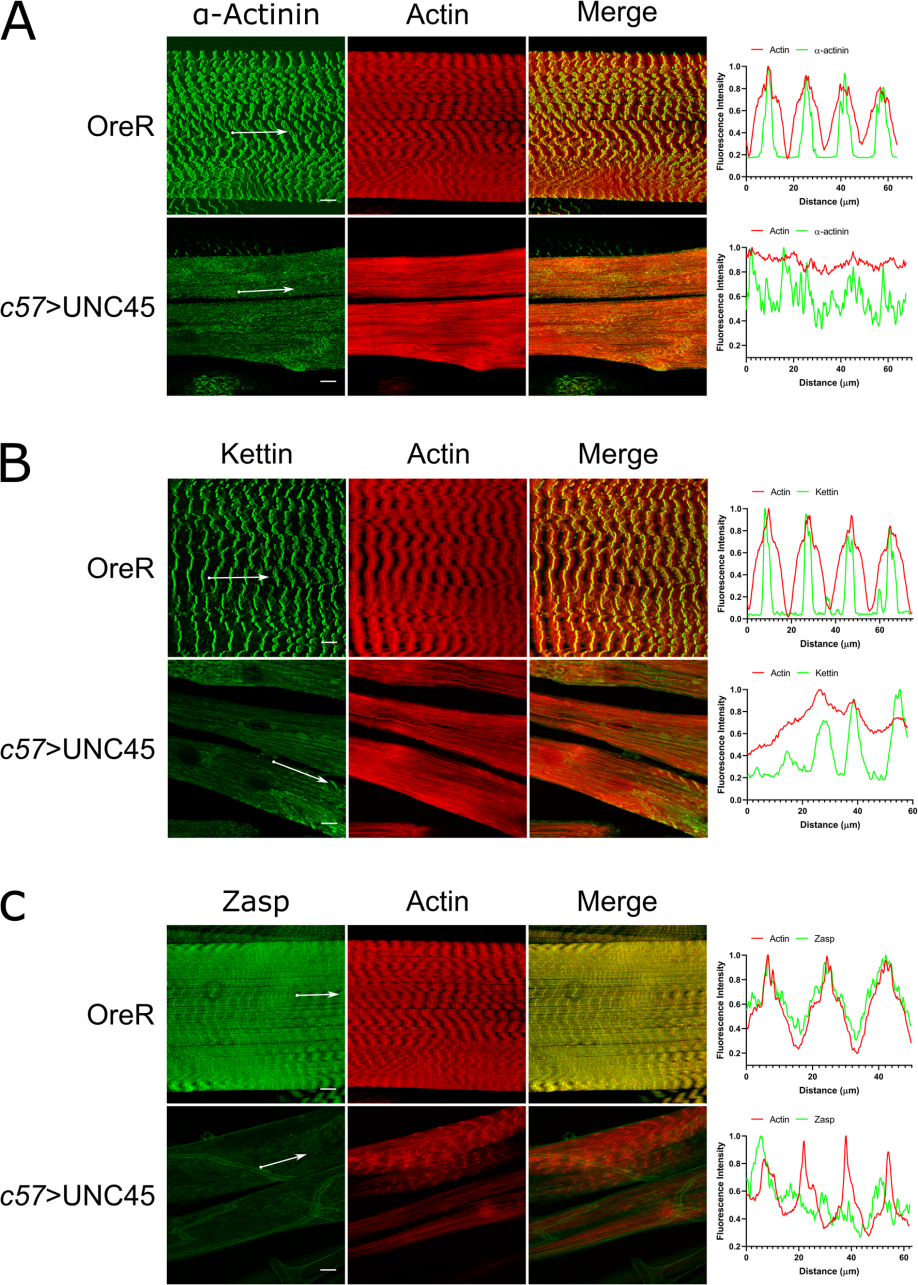
Changes in Z-disk structure in UNC45 knockdown larvae. Body wall muscles were labelled for **A**) actin (phalloidin) and α-actinin, **B**) actin and kettin or **C**) actin and Zasp. For each, the top panel shows the wildtype control animal and bottom panel show the c57 > UNC45 animals. Fluorescence intensity profiles on the right were taken along muscle fibrils indicated by the white arrows in the images on the left. Scale bar represents 10 μm

To further characterize the effects of the knockdown on sarcomere organization and Z-disk morphology, the larval body wall muscles were labelled for sarcomeric proteins (Fig. 4). Antibodies against α-actinin (Fig. 4A), kettin (Fig. 4B) and zasp (Fig. 4C) were used and co-stained with phalloidin. Kettin is a high molecular weight protein located within the I bands of *Drosophila* sarcomeres that binds to both actin and α-actinin and provides a link between thin and thick filaments [23, 24]. Adjacent actin filaments are anchored and cross-linked by α-actinin [25]. Zasp (Z-band alternatively spliced PDZ domain [26]) functions as a tension sensor and is involved in stabilizing the I-Z-I complex as well as recruiting α-actinin during sarcomere assembly [27, 28]. For all three proteins, a significant alteration in sarcomere structure was observed. The RNAi knockdown muscles exhibited no striations and the Z-lines appeared either discontinuous or irregular. The less severely affected muscles, that exhibited partial or disrupted banding patterns were observed to have both α-actinin and kettin localized in their respective spatial regions. However, their expression patterns were observed to be highly disorganized. Zasp was observed to be highly reduced in the I bands in any UNC45 knockdown muscles.

### Ultrastructural comparisons

**Fig. 5:**
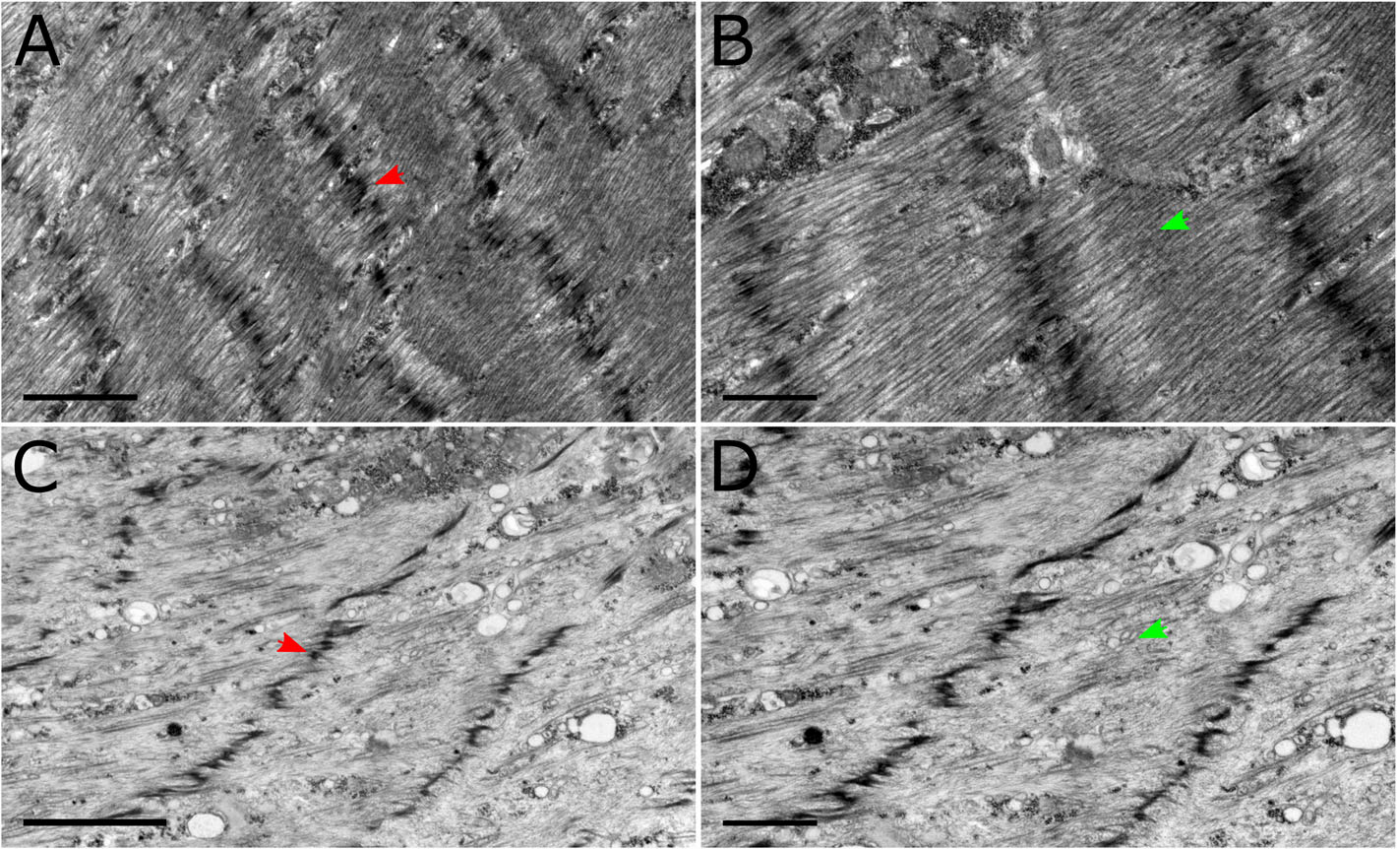
Transmission electron micrographs of larval body wall muscles. These are representative images of wildtype (**A**-**B**) and c57 > UNC45 larvae (**C**-**D**). Red and green arrowheads indicate Z-lines and M-lines respectively. Scale bars represent 2 μm (**A**, **C**) and 1 μm (**B**, **D**)

Ultrastructural analysis of larval body wall muscles was also performed using transmission electron microscopy (Fig. 5). Wildtype muscles showed highly organized sarcomeres with well-defined Z-disks (arrowhead in Fig. 5A) and M-bands (Fig. 5A and B). UNC45 knockdown muscles were found to have disruption in sarcomere structure, where we observed a reduction of myosin filaments in UNC45 knockdown muscle (Fig. 5C and D) compared to controls. Although present, the Z-disks were observed to be thinner with partial fragmentation, alongside sarcomeric disorganization (arrowhead in Fig. 5C) M-bands were not clearly defined as in the wildtype muscles.

### Synaptic physiology

**Fig. 6:**
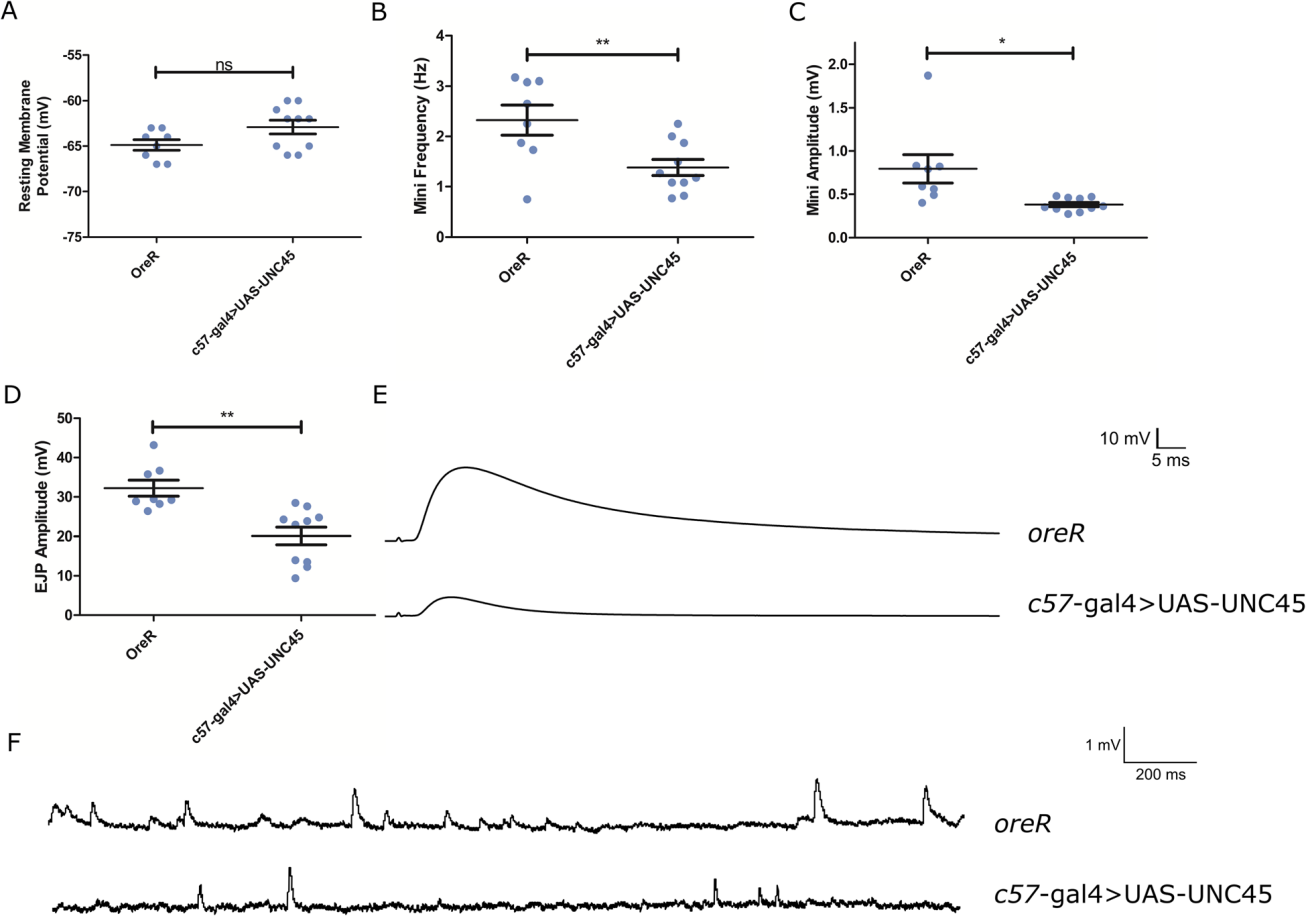
Electrophysiological data from UNC45 knockdown larvae. These are **A**) average resting membrane potential, **B**) average frequency for spontaneous release (±SEM), **C**) average amplitude of spontaneous release (±SEM), **D**) average nerve-evoked EJP amplitudes (±SEM) in HL3 buffer with 0.3 mM Ca2+, **E**) representative traces of nerve-evoked responses and F) Representative traces of spontaneous release events for both control and c57-Gal4 > UAS-UNC45

To further understand the extent and functional consequences in the UNC45 knockdown larvae, we next asked whether synaptic physiology was affected. We therefore recorded intracellular potentials from larval abdominal muscle 6. Interestingly, despite what appears as significant disorder of the internal structure, the muscle fiber resting membrane potential was similar in both knockdown and controls (Fig. 6A): in UNC45 knockdown larvae it was − 62.90 ± 2.37 mV and in wildtype controls it was − 64.87 ± 1.64 mV. This result suggests that at this level, the passive membrane properties of the muscle cell are not affected by the underlying sarcomeric disorder.

We next asked whether synaptic function was disrupted. First, spontaneous vesicle release was recorded and miniature excitatory junction potential (mEJP) amplitude (Fig. 6B) and frequency (Fig. 6C) were calculated. We observed that UNC45 knockdown larvae had a significant decrease in mEJP frequency and smaller mEJPs compared to control. The average mEJP frequency and amplitude for wildtype was found to be 2.33 ± 0.11 Hz and 0.79 ± 0.06 mV, whereas the average mEJP frequency and amplitude for *c57-Gal4* > UAS-UNC45 was found to be 1.38 ± 0.05 Hz and 0.38 ± 0.01 mV respectively.

Next, nerve-stimulated excitatory junction potentials (EJPs) were recorded and the average EJP amplitude was calculated (Fig. 6A and B). The UNC45 knockdown larvae had smaller EJP amplitudes compared to wildtype controls, where the EJPs recorded in 1 mM external Ca^++^ were found to be 32.2 ± 0.7 mV in control and 20.1 ± 0.7 mV in knockdown larvae.

Using this data for transmitter release, and applying the modified correction factors to calculate to quantal content (*m)* for *m=* $v^{\prime }/{v}_1\ \mathrm{and}\ {v}^{\prime }=\overline{v}/\left(1-f\left(\overline{v}/E\right)\right)$ according to Martin (1976) and McLachlin and Martin (1981) [29, 30], we find the quantal content of release in the UNC45 knockdown larvae is on average 55.0 quanta compared to 43 for wildtype controls. Despite the smaller measured EJP, this analysis suggests a strong physiological response is occurring to maintain some level of synaptic transmission at the neuromuscular junction.

### Locomotor behaviour

**Fig. 7:**
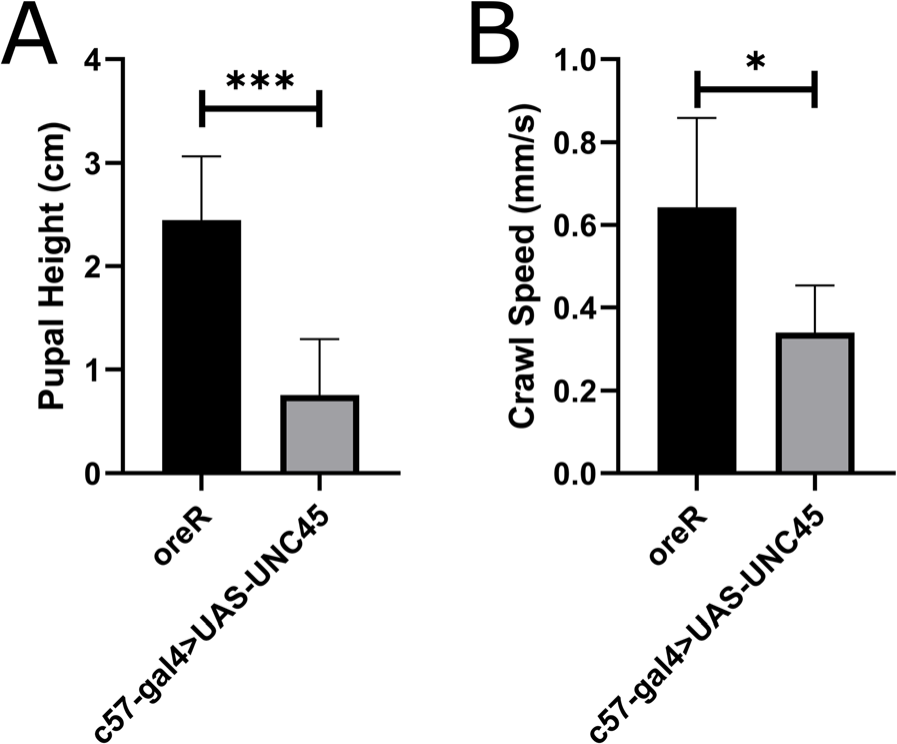
Effect of UNC45 knockdown on larval locomotion. Graphs depict **A**) Average pupation height (*n* = 75) and **B**) Average larval crawl speed (*n* = 15)

The larva’s ability to move through food and find a suitable pupation site could be affected by the UNC45 knockdown (Fig. 7). The distance between, the tail end of the pupal case and food was measured for 75 pupal cases for each (Fig. 7A). Qualitatively, we observed that UNC45 larvae often pupated close to or on the food media. When measured, we found that there was a significant decrease in pupation height in UNC45 knockdown larvae (0.76 ± 0.5 cm) compared to wildtype (2.44 ± 0.62 cm).

In addition, larval crawling speed was also investigated (Fig. 7B). Larval movement along an agar plate was recorded for 2 min, using 6 day old larvae and the average speed was tracked. It was observed that there was a significant decrease in the crawl speed of UNC45 (0.33 ± 0.12) knockdown larvae compared to wildtype (0.64 ± 0.17). Overall, UNC45 knockdown was found to negatively impact larval speed as well as ability to crawl vertical distances.

## Discussion

Muscle cells are highly specialized to generate force by contraction. This capacity allows muscle to power locomotion, to generate pressure, and to regulate the flow through tubular networks as observed with cardiac muscle. The ability to contract depends on the exquisite assembly of subcellular proteins into highly organized arrays of filaments, most notably myosin and actin filaments. In striated skeletal muscle, the initial assembly of protein filaments into sarcomeres and the subsequent maintenance of the structure depends on supporting protein chaperones to help guide molecular assembly and prevent inappropriate misfolding and aggregation. Among the group of chaperones, UNC45 has been identified as a key biochemical partner of myosin, alongside Hsp90 and Hsp70, to ensure proper incorporation of myosin monomers into characteristic myosin array of a sarcomere [11]. Here we focus on the role of UNC45 in striated muscle, but it should be noted that members of the broader UNC45 family also facilitate the function of non-muscle myosin in a variety of cell types (reviewed in [31]).

Prototypical UNC45 was first described in *C. elegans* [32] and orthologs are present in organisms from yeast to humans, including *Drosophila.* Research in key organisms such as *C. elegans* and *Drosophila* have helped to elucidate the function of UNC45 through molecular genetic analysis. A key advance in this study is that we found viable larvae with RNAi knockdown by screening three different Gal4 drivers. Two were embryonic lethal, but one, c57-Gal4, was late pupal lethal, which allowed examination of the earlier larval stages. Therefore, we could work with the easily accessible and very well characterized body wall muscles of the third instar larvae for both muscle structure and neuromuscular physiology.

Knockdown of UNC45 in muscle resulted in disruption of muscle sarcomeres in third instar larvae, as previously reported for embryonic body wall and cardiac muscle in *Drosophila* [14] and striated muscle of *C. elegans* [32]*.* We observed both abnormal banding pattern and reduced accumulation of myosin, consistent with prior reports [13]. We additionally examined and provide new data on three Z-disc proteins, to better understand subcellular structure. For each of α-actinin, kettin and Zasp, we observed mislocalization of these labeled proteins compared to control muscle, indicating a substantial breakdown of the underlying architecture. The immunofluorescence data were largely confirmed by electron microscopic images, in which we observed poorly organized sarcomeres and disorganized Z-discs. Altogether the imaging data indicate a significant disorganization of the normal sarcomeric structure of the larval body wall muscle. Notably, while actin filaments seem to maintain an orientation parallel to the long axis of the muscle, the I-Z-I structure is mainly lost, suggesting that incorporation and/or maintenance of myosin in the sarcomere array of protein filaments is necessary for proper sarcomere organization. Disruption in the sarcomeric structure due to changes in the expression levels a single protein has been shown before. For example, disruption of α-actinin localization was observed to have a similar effect in *C.elegans* [33]*.* Hence, the findings in this study strongly suggest that UNC45 plays a role in both sarcomere maintenance and the role of myosin organization in order to maintain structural integrity of the sarcomere cytoskeleton.

We did note incomplete penetrance of the phenotype. We measured 50% of muscle being severely affected, 25% partially affected and 25% no or little affect. Currently, we do not know the source of this variation, but we suspect some unevenness of Gal4 driver expression may underlie this observation.

Our physiological analysis revealed that while the passive membrane properties of the muscle were not affected by UNC45 knockdown, despite the underlying disorganizing, we found major changes in synaptic transmission. Both spontaneous mEJP amplitude and frequency were reduced. Traditionally smaller mEJPs are interpreted as a postsynaptic effect and there may be disruption of the glutamate receptors that respond to released neurotransmitter and indeed the phenotype we report is very similar to those in which direct manipulation of glutamate receptor subunits is examined [34–36]. Reduced mEJP frequency is often associated with presynaptic alterations and could reflect changes in release probability or active zone numbers. Further experiments to delineate these alternatives are required.

The evoked EJPs in UNC45 larvae (~ 20 mV) were substantially smaller than those in control samples (32 mV), likely owing to the 50% decrease in mEJP amplitude. However, in the context of smaller mEJPS, we calculated quantal content of evoked release to reveal that the UNC45 knockdown nerve terminals are releasing about 30% more quanta per impulse: 55.0 vs 43. Therefore, it seems that the UNC45 neuromuscular junction is mounting a substantial homeostatic response to maintain some functionality. Interestingly, in this situation the homeostatic response (reviewed in [37]) failed to maintain EJPs at normal levels whereas full compensation is found when synaptic strength reductions are forced by loss of glutamate receptors [34–36]. In some regards, our result is also similar to that found in *neto* mutants. Neto plays a role in glutamate receptor clustering and loss of *neto* also causes smaller mEJPs but there is apparently no homeostatic compensation [38]. Determining the underlying mechanisms of this synaptic response in the UNC45 knockdown will require examination of the known retrograde signaling pathways that contribute to synaptic homeostasis [37, 39]. In other cell types, UNC45 family members act as a chaperone for proteins other than myosin; it may be that UNC45 additionally interacts with one or more components in the retrograde signaling pathway. If so, knockdown of UNC45 could compromise a full and complete homeostatic response.

Part of our motivation to conduct this work is to advance our program of using non-linear microscopy to characterize biological tissue. Muscle is particularly suitable for second-harmonic imaging, arising from the non-centrosymmetric characteristics of the myosin filaments. Our prior studies have focused on wild type *Drosophila* muscle, including delineating structural properties and dynamics of contraction [16, 17]. This report is the first to apply SHG imaging to a mutant with the potential to disrupt the structure of larval muscle and confirms that SHG is a viable tool to detect muscle defects using a technique that requires no staining or other applied contrast agents. Thus, SHG technology may be particularly useful in future applications to detect or characterize muscle disease, from human muscle biopsy, for example.

## Conclusion

In this study we report UAS/Gal4-mediated knockdown of UNC45 in Drosophila third instar larva. Our results confirm the known role of UNC45 for myosin localization within the muscle sarcomere and extend these data to show the I-Z-I complex is also disrupted, suggesting the need for UNC45-dependent incorporation or maintenance of myosin for sarcomere integrity. Further, the ability to conduct this work in the well-characterized and accessible third instar allowed us to execute SHG and electron microscopy, alongside analysis of locomotion and synaptic physiology. At the larval neuromuscular junction, UNC45 knockdown led to significant changes in synaptic strength and revealed a partial compensation, likely due to homeostatic signaling to maintain synaptic transmission. Future work with UNC45 knockdown is likely to reveal more complex interactions required for muscle structure and function.

## Methods

### Drosophila genetics and dissections

All *Drosophila melanogaster* strains were maintained on Bloomington standard cornmeal at room temperature. Crosses and strains that produced larva for experimentation were raised at 25 °C. Oregon-R (OreR) was used as the wildtype control. The UAS/Gal4 system was used for tissue-specific knockdown of UNC45 expression. Three driver lines were tested: mef2-Gal4 expresses in the mesoderm in embryogenesis, 24B-Gal4 and c57-Gal4 expresses in muscles throughout the fly life cycle. To knockdown UNC45 expression, RNA interference (RNAi) was utilized. UAS-RNAi lines were obtained from Vienna Drosophila Resource Centre and tested (stock numbers 33,561). The progeny that were used were C47-Gal4/+; UAS-UNC45RNAi/+. Each cross consisted of 10 virgin females and 7 males.

To visualize the body wall muscles, wandering third instar larvae were dissected along the dorsal midline in zero calcium HL3 solution [40]. Upon removal of internal organs and fat bodies, the body was unfolded to expose the wall muscles and was fixed for 25 min in 4% formaldehyde in phosphate buffered saline (PBS). The tissue underwent several washes in fresh PBS before preparing for microscopic imaging. The fixed muscles were sandwiched in PBS between a 1 mm-thick microscope slide and a 0.17 mm-thick coverslip (No. 1.5) and sealed with nail polish.

### Antibody staining and confocal microscopy

After fixation the samples were washed in PBT (PBS containing 0.1% Triton X-100). The samples were blocked in 2% normal goat serum in PBT overnight at 4 °C. The primary antibodies used were rabbit anti-UNC45 (1:500, a gift from Sanford Bernstein), mouse anti-α-actinin (1:200, Development Studies Hybridoma Bank (DSHB)), mouse anti-myosin (1:200 DSHB), mouse anti-kettin (1:200 DSHB) and mouse anti-zasp (1:200, DSHB). The tissue was washed with PBT before incubating the samples in secondary antibodies for 2 hours at room temperature. Rhodamine-phalloidin (1:1000 Invitrogen) was used to visualize actin. Secondary antibodies used were Alexa Fluor 488 goat anti-mouse, 1:400 and Alexa Fluor 488 donkey anti-rabbit. The stained preparations were then mounted in Vectashield. Images were collected using a Zeiss LSM880 confocal microscope, using a 10× 0.3NA air objective and a 25× 0.8NA oil objective. All images were processed using ImageJ and Inkscape.

### Electron microscopy

*Drosophila* third instar larvae were dissected and fixed as described above. Sample embedding and sectioning were completed at Nanoscale Biomedical Imaging Facility, The Hospital for Sick Children, Toronto, Canada. The samples were fixed in 2% paraformaldehyde + 2.5% glutaraldehyde in 0.1 M sodium cacodylate buffer for 2 h at room temperature. The samples were then washed in 0.1 M sodium cacodylate buffer with 0.2 M sucrose, fixed for 1.5 h on ice in 1% osmium tetroxide and washed in cacodylate buffer. Samples were then dehydrated in an alcohol series, washed three times in 100% ethanol and then washed twice in propylene oxide. Samples were then infiltrated for 2 h in a mixture of 50:50 propylene oxide/Quetol-Spurr resin, then 100% resin for 2 h, before embedding them in 100% resin overnight at 65 °C. Samples were sectioned longitudinally, collected on mesh grids and stained with lead citrate and uranyl acetate. Images were collected in a FEI Technai 20 electron microscope.

### Nonlinear optical microscope and SHG measurements

Samples for SHG imaging were dissected as described above and mounted in PBS solution.

Imaging and measurements were taken with a custom-built single-photon counting multi-contrast nonlinear laser scanning microscope [22]. The laser source consisted of an extended cavity femtosecond ytterbium-doped potassium gadolinium tungstate (Yb:KGd(WO_4_)_2_ or referred to as Yb:KGW) crystal based oscillator that provides 450 fs duration pulses at wavelengths centered at 1028 nm and a pulse repetition rate of 14.3 MHz [41]. The microscope setup, along with the excitation pathway is shown in [42]. The objective used for all the experiments was a 20× 0.75 NA air objective (Zeiss) with a working distance of 600 μm. The SHG signal was collected using a custom 0.85 NA collection objective and filtered using a band pass interference filter for 510 ± 10 nm and BG39 color glass optical filter, and detected by a photon-multiplier tube (PMT) (H7421–40, Hamamatsu).

### Electrophysiology

Intracellular recordings were made using an AxoClamp 2B amplifier (Molecular Devices, Sunnyvale CA). An electrode filled with 3 M KCl was inserted into muscle 6 at hemi-segments A3 or A4. The severed segmental nerve was stimulated using a suction electrode filled with saline. Miniature excitatory junction potentials (mEJPs) were recorded for 1.5 mins. Nerve-stimulated potentials (EJPs) were recorded at 1 Hz. All analysis was done using Clampfit 10.0 (Molecular Devices, Sunnyvale CA).

### Behaviour assays

For pupation height assays, eggs were collected in 4-h windows and placed in vials of fresh medium at 25 °C for 3–4 days. Larvae were then picked out of the vial and place in fresh food. This was to avoid discrepancies in height measurements due to food liquefaction. Approximately 25 larvae were placed in each vial. Pupal height measurements were done after. The food surface was marked as zero and pupation height was measured as the distance between food surface to the posterior end of the pupal case. Pupal case found to be in contact with the food surface was recorded as zero.

For larval locomotion assays, eggs were collected in 4 h windows in vials and aged at 25 °C for 6 days. After the incubation period, 3rd instar larvae were picked out of the vial individually and placed on a petri dish with grape juice agar and movement was recorded using an iPhone XR camera (Apple, CA) for 3 mins. Larval crawl speed was measured using Noldus Ethovision XT (Noldus Information Technology, Netherlands).

### Statistics

All statistical analyses were performed using Prism (v8.0, GraphPad, Software, La Jolla, CA, USA). Student’s *t* test was used two-way comparisons and ANOVA analysis and Tukey-Kramer post-hoc test was used for comparisons with more than two datasets. Throughout the text, data are reported as mean ± standard error of the mean (±SEM). On the relevant figures, statistical comparison of RNAi samples vs control are indicated as ***p < 0.0005, **p < 0.005, *p < 0.05 and ns = not significant.

## Data Availability

The datasets used and/or analyzed during the current study are available from the corresponding author on reasonable request.

## Abbreviations

EJP: Excitatory Junction Potential
PMT: Photon Multiplier Tube
SEM: Standard Error of the Mean
SHG: Second Harmonic Generation
TPR: N-terminal Tetratricopeptide Repeat
Zasp: Z-band alternatively spliced PDZ domain
